# Editorial: Plant biotechnology and genetics for sustainable agriculture and global food security

**DOI:** 10.3389/fpls.2024.1479632

**Published:** 2024-08-30

**Authors:** Yinglong Chen, Baohong Zhang, Galal Bakr Annis

**Affiliations:** ^1^ School of Agriculture and Environment, and UWA Institute of Agriculture, The University of Western Australia, Perth, WA, Australia; ^2^ Department of Biology, East Carolina University, Greenville, NC, United States; ^3^ Field Crops Research Institute, Agricultural Research Center, Giza, Egypt

**Keywords:** plant biotechnology and breeding, genetics, genome-wide association, next generation sequencing, CRISPR, omics, agriculture sustainability, genotyping (GT)

The global landscape of food security is increasingly precarious, exacerbated by multifaceted challenges ranging from conflict-driven crises to the compounding impacts of climate change and economic instability. According to the World Food Programme, people facing acute hunger has nearly doubled since 2019, from 135 million to 258 million, a number that could potentially double again due to the disruptive effects of the COVID-19 pandemic, placing an additional 130 million at risk (Ramanujam et al., 2024). Moreover, pervasive food insecurity not only jeopardizes public health with its links to malnutrition-related illnesses and premature deaths but also underscores a critical need for enhanced agricultural productivity.

Since the inception of the Green Revolution, strides in crop productivity have been significant but insufficient. Annual yield increases for major crops currently hover between 0.8% to 1.2%, a pace that falls short of what is required to sustainably feed a burgeoning global population (Ahmad, 2023). Classical breeding techniques have historically played a pivotal role in enhancing crop varieties and ensuring food security. However, their limitations in meeting contemporary global demands have spurred a drive towards novel plant breeding techniques (NPBTs) and other advanced agricultural methodologies.

In recent years, breakthroughs such as genome-wide association studies (GWAS), Next Generation Sequencing (NGS), and genome editing (GE) have emerged as transformative tools in crop improvement efforts (Mangrauthia et al., 2024). These technologies offer unprecedented access to vast gene pools, enabling targeted enhancements in traits essential for crop resilience, yield, and nutritional quality. Versatile techniques, such as Clustered Regularly Interspaced Short Palindromic Repeats (CRISPR)/CRISPR-associated protein (CRISPR/Cas) genome editing, marker-assisted selection, and Quantitative Trait loci (QTL) mapping exemplify the precision and potential of NPBTs to fortify crops against various environmental biotic and abiotic stresses while optimizing productivity.

This Research Topic explores cutting-edge research in novel plant breeding strategies, with a focus on bolstering crop tolerance to abiotic and biotic stresses. It elucidates the mechanistic underpinnings of gene function and regulation with the advancements in CRISPR/Cas-based editing techniques and integrative multi-omics analyses.

## Advanced plant biotechnology and genetics approaches

Traditional methods in quantitative genetics, including GWAS, genomic selection, QTL mapping, have historically formed the backbone of crop breeding programs (Maldonado et al., 2020; Thudi et al., 2021). Compared to Bayesian approaches, the efficacy of deep learning models for predicting flowering-related traits in a tropical maize panel from Brazil using single nucleotide polymorphisms (SNPs) data across multiple environments was evaluated in a study of Mora-Poblete et al. Multi-trait models showed a 14.4% increase in prediction accuracy over single-trait models, while multi-environment schemes improved accuracy by 6.4%. Deep learning consistently outperformed Bayesian methods in both scenarios. Genome-wide association analysis identified significant marker-trait associations, highlighting the potential of deep learning in enhancing genomic selection for improving maize breeding programs focused on flowering traits.

The challenge of maintaining crop health in the face of evolving pathogens is outlined in a review of Yıldırım et al., which emphasizes the limitations of traditional breeding methods against emerging diseases exacerbated by climate change. It underscores the promising role of genome editing in enhancing crop resilience by targeting defense-related genes, thereby advancing sustainable agriculture and bolstering food security through durable disease resistance strategies. The study advocates for genome editing as a pivotal tool in realizing the concept of “healthy plants” capable of withstanding diverse biotic stresses.

Using k-mer-based sequence comparison and deep learning-based variant calling approaches, Ruperao et al. investigated genetic and morphological variation in sorghum race populations. The analysis of 272 sorghum accessions revealed 1.7 million high-quality SNPs and identified 2,370 genes associated with selection signatures, including 179 selective sweep regions across 10 chromosomes. These findings highlight the genetic basis of domestication traits such as biomass and plant height, with implications for future plant breeding programs.

Through computational analysis and expression profiling technologies, Tan et al. identified 37 potassium (K^+^) transport-related genes (PTGs) in mango (*Mangifera indica*), including 22 K^+^ transporters and 15 K+ channels, essential for plant growth, development, and drought tolerance. Phylogenetic and promoter analyses revealed strong kinship with other plants and identified cis-elements related to various biological processes. RNA-seq expression profiling highlighted specific PTGs upregulated in roots and leaves and during different growth stages, providing foundational knowledge for understanding K^+^ transport in mango and aiding in the functional characterization of K^+^ genes in tropical fruits.

In a review of Das et al., the authors highlight that plants are a crucial source of specialized metabolites, offering physiological benefits and evolutionary advantages, especially in defence mechanisms. Advances in transgenic techniques like gene silencing and overexpression have boosted the production of these compounds, reducing costs and enhancing nutritional value. The use of CRISPR/Cas-based gene editing is now pivotal in improving the yield of specific metabolites in medicinal plants, offering significant advancements in metabolic engineering for future applications.

## Plant biotechnology and genetics reveal plant responses to environmental stress

A study reported in Geldhof et al. investigates how tomato plants adapt to waterlogging through downward leaf bending, a trait influenced by complex genetic and environmental interactions. Using a GWAS of 54 tomato accessions, researchers identified candidate genes linked to plant survival under waterlogged conditions. The study highlighted genes potentially involved in metabolic adjustments and leaf angle dynamics, suggesting their roles in facilitating plant resilience and recovery from waterlogging stress.

In the review of Devi et al. the deficiency of methionine in maize, a critical amino acid essential for animal and human nutrition, is addressed. This review explores biofortification strategies, emphasizing the role of zein proteins, particularly δ-zein, in methionine deposition within maize kernels. The study consolidates various approaches including natural mutant selection, genetic engineering techniques, and meta-QTL studies to enhance methionine content in maize, offering insights for future research aimed at developing high-methionine maize varieties to improve nutritional outcomes sustainably.

Shang et al. identified and characterized 82 class III peroxidase (PRX) proteins in sugarcane, revealing their role in lignification, cell elongation, seed germination, and stress responses. Phylogenetic and promoter analyses showed the evolutionary history and regulatory elements of ShPRX genes, highlighting their response to ABA, MeJA, light, anaerobic conditions, and drought. Differential expression and qRT-PCR analyses demonstrated that these genes are specifically induced by SCMV, Cd, and salt, providing insights into their structure, evolution, and potential for developing stress-resistant sugarcane varieties and phytoremediation strategies.

Ma et al. summarised the multifaceted research on pennycress (*Thlaspi arvense* L.), an emerging oil crop within the Brassicaceae family. The review highlighted several key research areas including its utility as a model plant akin to *Arabidopsis thaliana*, advancements in oil and protein extraction technologies, metabolomics-based seed composition analysis, germplasm development, ecological impacts, abiotic stress responses, and strategies for optimizing fatty acid extraction. Future research directions proposed include assembling the pennycress genome, refining biodiesel extraction processes, investigating molecular mechanisms of fatty acid synthesis, and elucidating the roles of pivotal genes under diverse environmental stresses.

Liu et al. investigated how different host plants influence the biological traits, nutritional metabolism, and *Buchnera* symbiont dynamics in the pea aphid (*Acyrthosiphon pisum*). It reveals significant variations in *Buchnera* titers across host plants, with higher levels observed on *T. pratense* and *M. officinalis*. Pea aphids on broad bean (*Vicia faba*) exhibited increased levels of soluble sugar, glycogen, and total energy, correlating with enhanced fecundity and weight. These findings underscore the complex interplay between host plant species, symbiotic interactions, and nutritional outcomes in aphid populations, offering insights into broader implications for insect development and management strategies.

Travadi et al. validated rbcL and ITS2 metabarcoding primers for detecting plant species in herbal products, using mock controls of medicinal plant DNA and biomass pools. This study demonstrates high detection efficiencies of 86.7% and 71.7% for rbcL, and 82.2% and 69.4% for ITS2, in DNA and biomass pools respectively. Combining these metabarcodes achieved a cumulative detection efficiency of 100% in DNA pools and 90% in biomass pools. Overall, the study underscores the potential of multilocus metabarcoding as a robust tool for identifying labelled and unlabelled plant species in herbal formulations, thereby enhancing pharmacovigilance in the global herbal medicine market.

## Conclusions

Recent advancements in plant biotechnology and genetics underscore a transformative era in crop improvement and sustainable agriculture. Traditional methods such as GWAS, genomic selection, and QTL mapping continue to play pivotal roles in enhancing crop breeding programs, facilitating targeted trait improvements across diverse environments. The integration of deep learning models has shown promising results, thereby enriching the precision and efficiency of genomic selection. Furthermore, innovations in genome editing technologies offer new avenues for enhancing crop resilience against evolving pathogens and environmental stresses, contributing to global food security. Studies in this Research Topic highlight the evolving landscape of plant biotechnology, emphasizing the critical intersection of genetics, computational analysis, and biotechnological tools in shaping the future of agricultural sustainability and productivity.
